# Supplementary figures and images for: Evolutionary couplings detect side-chain interactions
Source: PeerJ. 2019 Jul 8;7:e7280. doi: 10.7717/peerj.7280 (PMC6622159; doi:10.7717/peerj.7280)

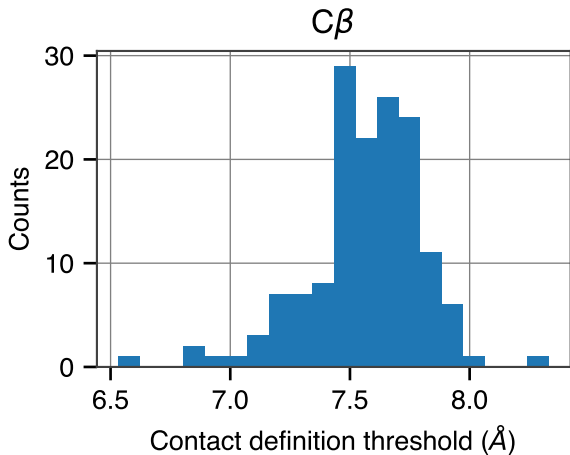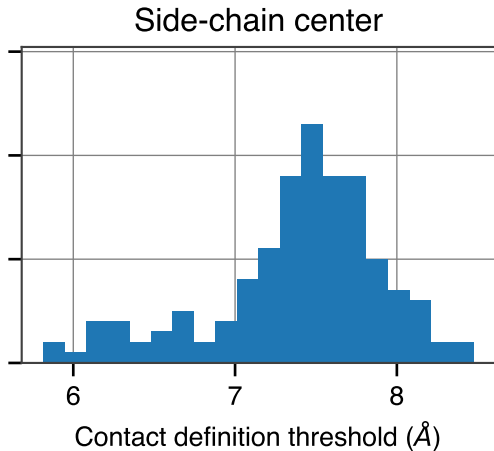

Supplement: Supplemental Information 1 — To directly compare contact identification metrics, contacts for a given protein were first defined according to Cα atoms with an 8 Å distance cutoff. For Cβ (left) and side-chain center (right), an equal number of putative contacts were then identified regardless of their distances. Histograms show the relevant distance cutoffs that ensure an equal number of putative contacts for each metric for the set of 150 proteins. [file peerj-07-7280-s001.pdf]

**A**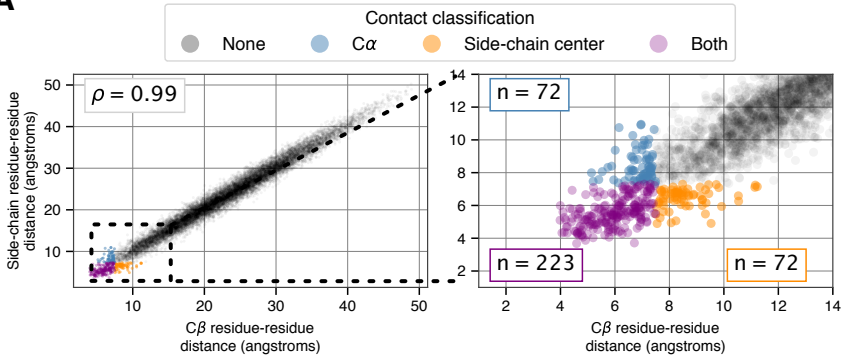**B**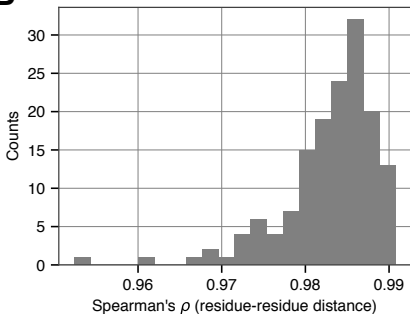**C**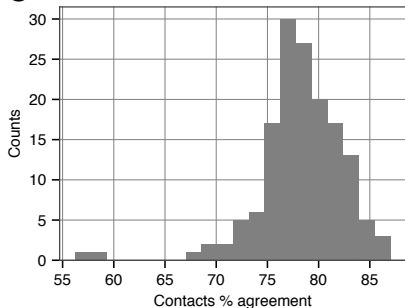

Supplement: Supplemental Information 2 — Similar to Figure 2; here, we compare residue–residue distances defined via Cβ and side-chain centers. (A) For PDB:1AOE, Cβ and side-chain center contacts were defined according to 7.5 A and 7.33 A distance cutoffs, respectively. (B) Distribution of Spearman’s correlation coefficient values (ρ) between residue–residue distances for 150 different proteins. (C) Distribution of the percent agreement for contact definitions for the same set of proteins. [file peerj-07-7280-s002.pdf]

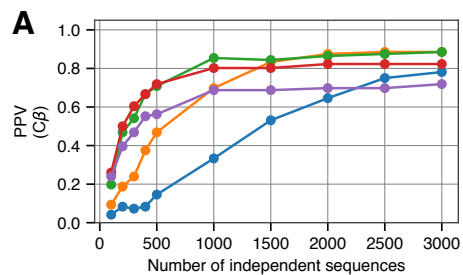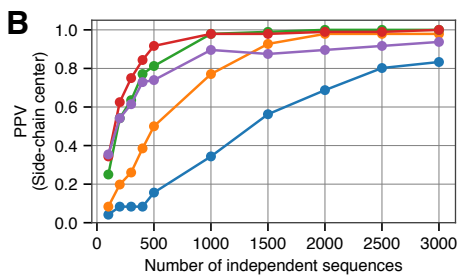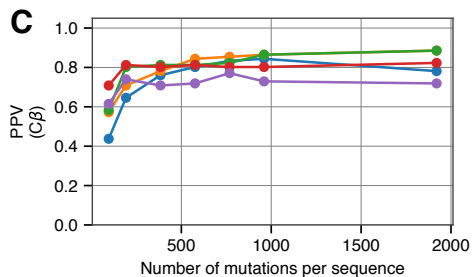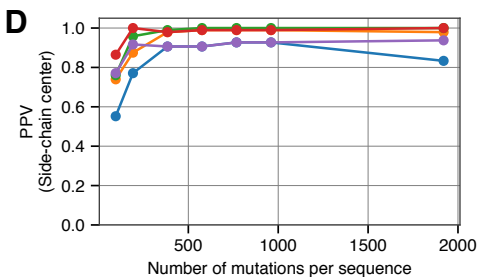

Supplement: Supplemental Information 3 — Similar to Figure 3; here, we compare Cβ and side-chain center contact definitions. (A) For each of 5 separate selection strengths (colored lines), we ran simulations until a number of mutations totaling 10 times the length of the protein were accumulated per replicate. We varied the number of independent replicate sequences (x-axis) that were used as input for evolutionary coupling analysis, and found that couplings fail to fully recover Cβ defined contacts for PDB:1AOE. (B) By contrast, contacts defined via side-chain centers are near-perfectly recovered across a range of simulation parameters. (C) and (D) Similar to parts (A) and (B), but along the x-axis we now show results from simulations where a different number of accepted mutations were accumulated per sequence. We fixed the number of replicate sequences that were simulated—and used for evolutionary coupling analysis—at 3,000 for each of these data points. (note: side-chain center plots—(B) and (D)—are the same as in Figure 3 and are included here only for comparison.). [file peerj-07-7280-s003.pdf]

Spearman's  $\rho = 0.669$ 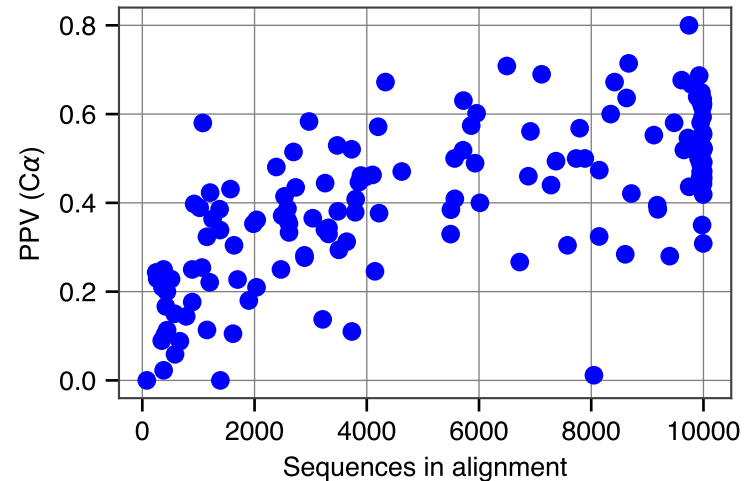Spearman's  $\rho = 0.681$ 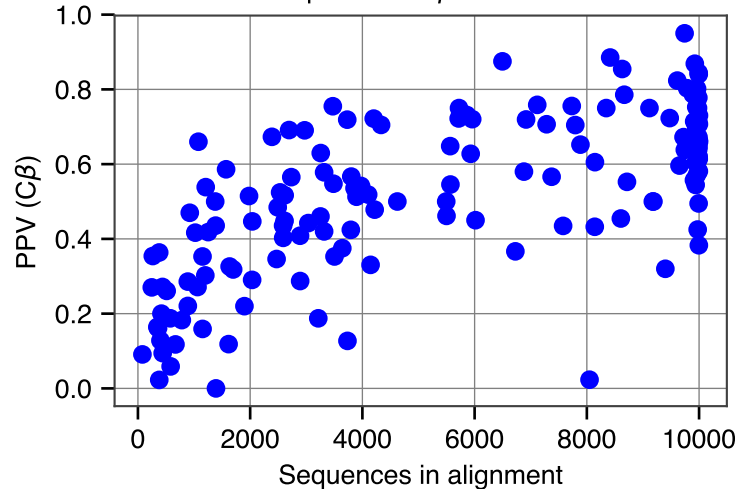Spearman's  $\rho = 0.684$ 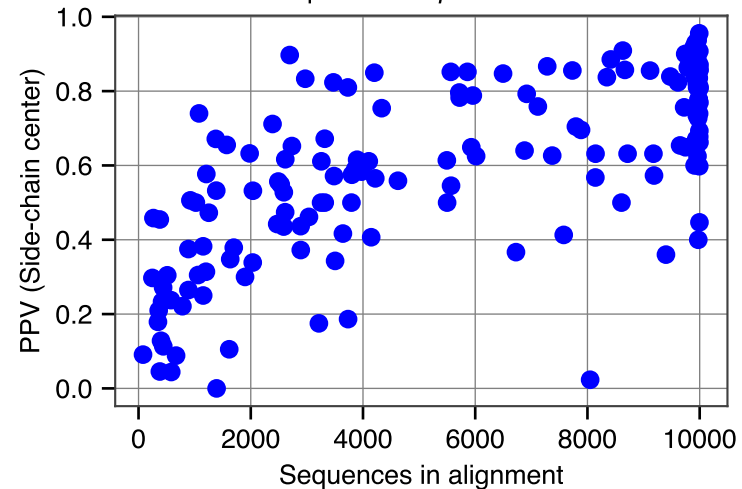

Supplement: Supplemental Information 4 — PPVs for all three different contact identification methods (y-axes) are strongly correlated with the number of sequences in the empirical sequence alignments used as input for calculating evolutionary couplings. PPV data here is the same data from Figure 4. [file peerj-07-7280-s004.pdf]

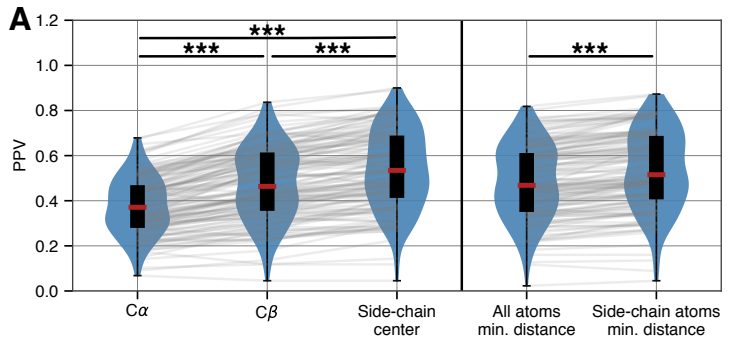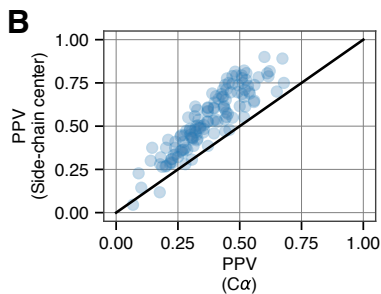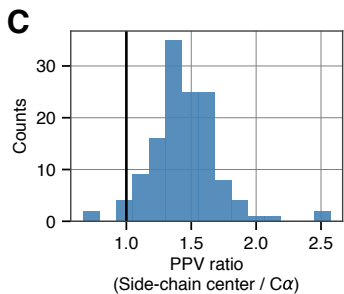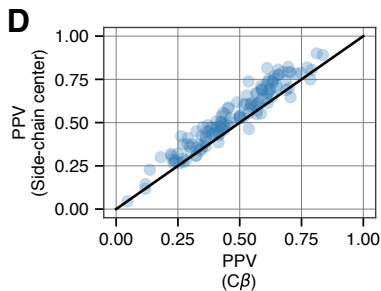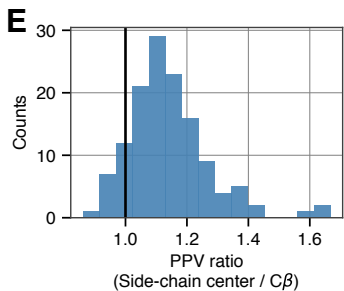

Supplement: Supplemental Information 5 — Similar to Figure 4; here, we use the PSICOV method to calculate evolutionary couplings. (A) For a diverse set of 150 proteins, the PPV of the top L/2 evolutionary coupling scores—derived from empirical sequence alignments—is progressively higher when intramolecular contacts are defined according to Cα atoms, Cβ atoms, and side-chain centers. Similarly, PPVs are higher when computing contacts based on side-chain atoms only as opposed to considering all possible interactions between atoms in residues. (*** indicates p < 10−20, Wilcoxon signed-rank test) (B) Scatter plot of PPVs for each protein according to Cα and side-chain center contact identification methods. (C) Histogram of the ratios from the data in (B) to estimate the effect size. (D) and (E) As in (B) and (C), comparing Cβ and side-chain center contact identification methods. [file peerj-07-7280-s005.pdf]

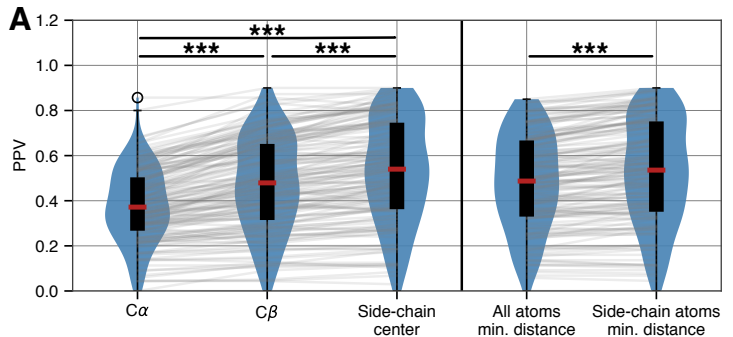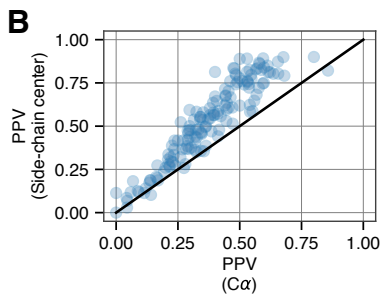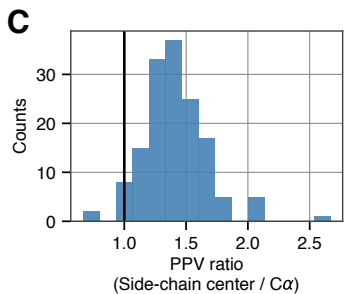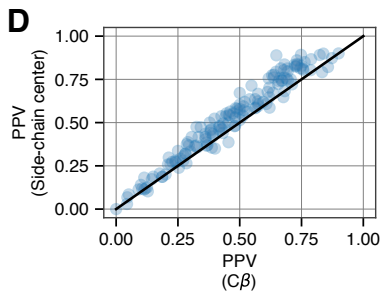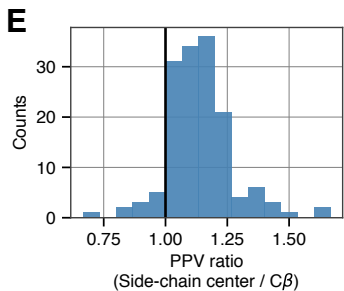

Supplement: Supplemental Information 6 — Similar to Figure 4; here, we use the PLMC method to calculate evolutionary couplings. (A) For a diverse set of 150 proteins, the PPV of the top L/2 evolutionary coupling scores—derived from empirical sequence alignments—is progressively higher when intramolecular contacts are defined according to Cα atoms, Cβ atoms, and side-chain centers. Similarly, PPVs are higher when computing contacts based on side-chain atoms only as opposed to considering all possible interactions between atoms in residues. (*** indicates p < 10−20, Wilcoxon signed-rank test) (B) Scatterplot of PPVs for each protein according to Cα and side-chain center contact identification methods. (C) Histogram of the ratios from the data in (B) to estimate the effect size. (D) and (E) As in (B) and (C), comparing Cβ and side-chain center contact identification methods. [file peerj-07-7280-s006.pdf]

**A**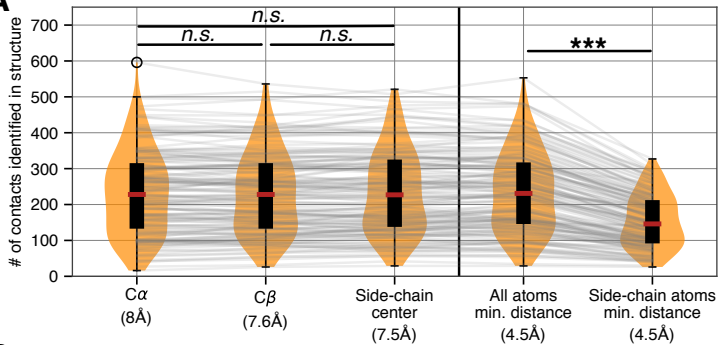**B**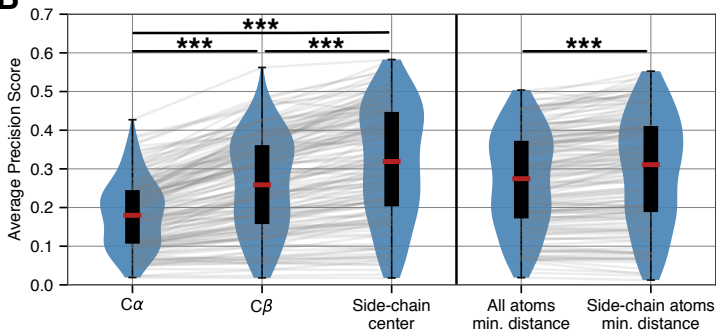

Supplement: Supplemental Information 7 — Using a fixed distance cutoff to determine contacts for each method illustrates heterogeneity in contact numbers and prediction accuracy. (A) The number of contacts defined in each of 150 proteins according to the method and the distance cutoff (in angstroms). (*** indicates p < 10−20, Wilcoxon signed-rank test; n.s.indicates p > 0.05). (B) Due in particular to the large difference in the number of contacts identified by the ‘Side-chain atoms min. distance’ method, we repeated the results of Fig. 4 using fixed cutoffs (as indicated in panel A) and the Average Precision Score as a metric of accuracy, which is more comparable than PPV across sets with varying numbers of true positives. (*** indicates p < 10−20, Wilcoxon signed-rank test). [file peerj-07-7280-s007.pdf]

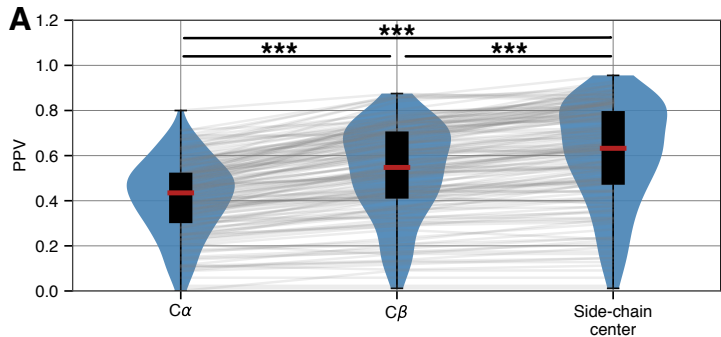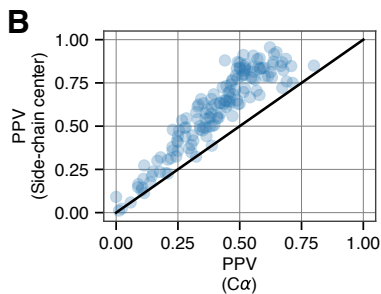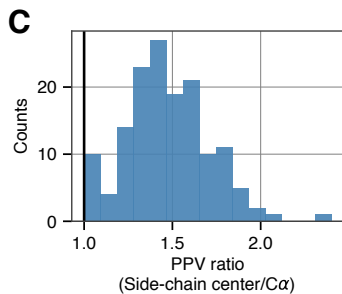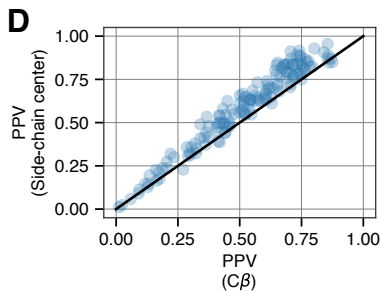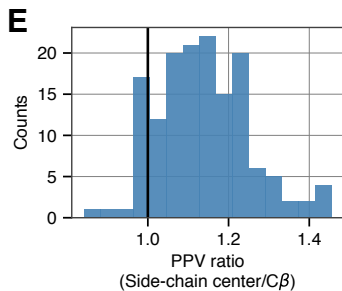

Supplement: Supplemental Information 8 — Similar to Figure 4; here, we show results when using a fixed distance cutoff to define contacts individually for the three separate methods: Cα (8A), Cβ (7.6A), side-chain center (7.5A). (A) For a diverse set of 150 proteins, the PPV of the top L/2 evolutionary coupling scores—derived from empirical sequence alignments—is progressively higher when intramolecular contacts are defined according to Cα atoms, Cβ atoms, and side-chain centers. (*** indicates p < 10−20, Wilcoxon signed-rank test) (B) Scatter plot of PPVs for each protein according to Cα and side-chain center contact identification methods. (C) Histogram of the ratios from the data in (B) to estimate the effect size. (D) and (E) As in (B) and (C), comparing Cβ and side-chain center contact identification methods. [file peerj-07-7280-s008.pdf]
